# Bilateral radial agenesis with absent thumbs, complex heart defect, short stature, and facial dysmorphism in a patient with pure distal microduplication of 5q35.2-5q35.3

**DOI:** 10.1186/1471-2350-14-13

**Published:** 2013-01-24

**Authors:** Aleksander Jamsheer, Anna Sowińska, Dorota Simon, Małgorzata Jamsheer-Bratkowska, Tomasz Trzeciak, Anna Latos-Bieleńska

**Affiliations:** 1 Department of Medical Genetics, University of Medical Sciences in Poznan, 55 Grunwaldzka Street, pav. 15, Poznan, 60-352, Poland; 2 NZOZ Center for Medical Genetics GENESIS, Poznan, 4 Grudzieniec Street, Poznan, 60-601, Poland; 3 National Institute of Public Health - National Institute of Hygiene, Department of Environmental Hygiene, 24 Chocimska Street, Warsaw, 00-791, Poland; 4 Department of Orthopedics and Traumatology, University of Medical Sciences, Poznan, 135/147 28 czerwca 1956 r. Street, Poznan, 61-545, Poland

**Keywords:** Pure distal trisomy 5q, Distal 5q duplication, Dup (5)(q35.2q35.3), Hunter-McAlpine syndrome, *MSX2*, *FGFR4*, Radial agenesis, Absent thumbs

## Abstract

**Background:**

A partial duplication of the distal long arm of chromosome 5 (5q35-- > qter) is known to be associated with a distinct phenotype referred to as Hunter-McAlpine syndrome. Clinical spectrum of this disorder mainly consists of mental retardation, microcephaly, short stature, skeletal anomalies, and craniofacial dysmorphism featuring flat facies, micrognathia, large, low-set dysplastic ears, hypertelorism, almond-shaped, down-slanted palpebral fissures, epicanthal folds, small nose, long philtrum, small mouth, and thin upper lip. Less frequent remarkable findings include craniosynostosis, heart defect, hypoplastic phalanges, preaxial polydactyly, hypospadias, cryptorchidism, and inguinal hernia. In most patients with a partial duplication of 5q the aberration occurred due to an inherited unbalanced translocation, therefore the phenotype was not reflective of pure trisomy 5q.

**Case presentation:**

We report on a 9.5-year-old boy with some feature of Hunter-McAlpine syndrome including short stature, complex heart defect (dextrocardia, dextroversion, PFO), bilateral cryptorchidism, hypothyroidism, and craniofacial dysmorphism. Additionally, bilateral radial agenesis with complete absence of Ist digital rays, ulnar hypoplasia with bowing, choroidal and retinal coloboma, abnormal biliary vesicle were identified, which have never been noted in 5q trisomy patients. Karyotype analysis, sequencing and MLPA for *TBX5* and *SALL4* genes were unremarkable. Array comparative genomic hybridization detected a duplication on 5q35.2-5q35.3, resulting from a *de novo* chromosomal rearrangement. Our proband carried the smallest of all previously reported pure distal 5q trisomies encompassing terminal 5.4-5.6 Mb and presented with the most severe limb malformation attributed to the increased number of distal 5q copies.

**Conclusions:**

We postulate that a terminal distal trisomy of 5q35.2-5q35.3, which maps 1.1 Mb telomeric to the *MSX2* gene is causative for both radial agenesis and complex heart defect in our proband. A potential candidate gene causative for limb malformation in our proband could be *FGFR4*, which maps relatively in the closest position to the chromosomal breakage site (about 1.3 Mb) from all known 5q duplications. Since the limb malformation as well as the underlying genetic defect are distinct from other 5q trisomy patient we propose that a position effect resulting in altered long-range regulation of the *FGFR4* (alternatively *MSX2*) may be responsible for the limb malformation in our proband.

## Background

A partial duplication of the distal long arm of chromosome 5 (5q35-- > qter) is known to be associated with a distinct phenotype referred to as Hunter-McAlpine syndrome [1]. Clinical spectrum of this disorder consists mainly of mental retardation, microcephaly, short stature, skeletal anomalies, and craniofacial dysmorphism featuring flat facies, micrognathia, large, low-set dysplastic ears, hypertelorism, almond-shaped, down-slanted palpebral fissures, epicanthal folds, small nose, long philtrum, small mouth, and thin upper lip. Less frequent remarkable findings include craniosynostosis, heart defect (VSD, ASD, bicuspid aortic valve), hypoplastic phalanges, preaxial polydactyly, hypospadias, cryptorchidism, and inguinal hernia [2-7]. In most patients with a partial duplication of 5q the aberration occurred due to an inherited unbalanced translocation, hence the phenotype was not reflective of pure trisomy 5q. To our knowledge, the smallest pure partial distal duplication of chromosome 5q described to date encompassed about 6.4 Mb of 5q terminus (5q35.2-5q35.3) and was detected in a patient presenting with microcephaly, strabismus, facial dysmorphism, moderate mental retardation, short stature, brachydactyly, and inguinal hernias [8]. Additionally, one more case with a pure gain on distal 5q (an interstitial triplication of 5q35.2-5q35.3) involving 6.56 Mb was identified in a patient manifesting some common features of Hunter-McAlpine syndrome (intrauterine growth retardation, almond-shaped eyes with epicanthal folds, downturned mouth with thin vermillion of the upper lip), as well as other unique findings such as left ventricular noncompaction (LVNC) and absent thumbs [9].

In our report, we describe a male proband with a pure 5.4-5.6 Mb distal duplication of 5q35.2-- > qter detected by array comparative genomic hybridization (array CGH), resulting from a *de novo* chromosomal rearrangement. To our knowledge, this is the smallest region of pure distal 5q trisomy. In addition to some features of Hunter-McAlpine syndrome, our patient presented with bilateral radial aplasia with absent thumbs, which seems to be the most severe limb malformation attributed to the increased number of distal 5q copies.

### Clinical report

**Figure 1:**
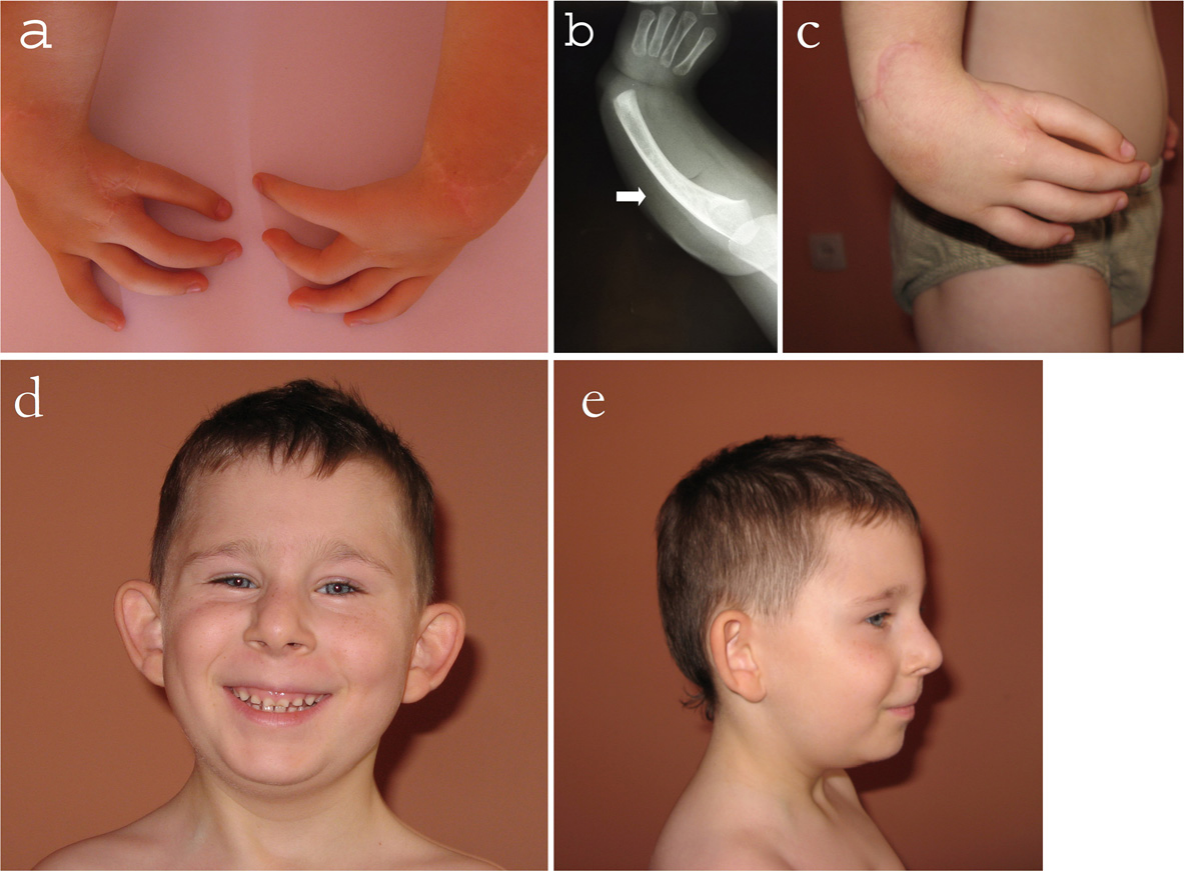
**The proband at the age of 8 years and 4 months: a**) Bilateral upper limb defect comprised of absent thumbs and radial bone agenesis resulting in significant forearm shortening **b**) X-ray scan of the proband’s left forearm showing absent radius along with Ist digital ray **c**) right forearm of the proband with prominent contraction of the zeugopod **d**) Facial dysmorphic features seen in the proband **e**) Lateral view of the proband’s face.

The proband, a 9.5-year-old boy of Polish descent, was born by spontaneous delivery after uneventful pregnancy (G1P1) at 38 weeks of gestation to a non-consanguineous and healthy 24-year-old mother and a 30-year-old father. At birth, his weight was 2500 g (3rd percentile), length 51 cm (75th-90th percentile), head circumference 32 cm (3rd percentile), and Apgar score was 9-10-10 at 1, 3, and 5 minutes, respectively. Physical examination after birth showed bilateral upper limb malformation comprised of radial agenesis with complete absence of Ist digital ray (i.e. absent thumbs as well as Ist metacarpals), and ulnar hypoplasia with bowing. Both forearms were significantly shortened due to the long bone aplasia/hypoplasia (Figure 1a, 1b and 1c). In addition, facial dysmorphism and bilateral cryptorchidism were noted. Echocardiography (ECHO) exam revealed dextrocardia, dextroversion, and persistent foramen ovale apertum (PFO). On abdominal ultrasound at 5 days of age an abnormally long inverted U-shaped biliary vesicle was noted. Hearing tests performed during first week after birth showed left hypoacusis. At the age of 4 months a detailed ophthalmologic examination revealed a congenital choroidoretinal coloboma in the down quarter of the right eye. The boy was referred to our genetic clinic for diagnosis and first investigated at the age of 7 years. Upon examination, he presented with short stature, neck webbing, and craniofacial dysmorphic features comprising full cheeks, prominent widened nasal bridge, almond-shaped eyes, thin vermillion of upper lip, abnormally shaped teeth, and low-set dysplastic ears (Figure 1d and 1e). His height reached 113 cm (below 3rd percentile), whereas body weight was 20 kg (10th percentile). According to anamnesis, developmental milestones were achieved on time, with independent sitting at 6, walking at 12, and expressive speech (first several words) at 12 months. Retrospective analysis of medical record revealed that short stature had become evident before 2 years of age: 7.8 kg (below 3rd percentile) and 80 cm (below 3rd percentile) at age 2 years. Due to facial dysmorphism and microsomia recognized at that time, the proband was referred for a conventional chromosomal analysis (performed on peripheral blood lymphocytes with a resolution of 550 bands per haploid genome) which showed normal male karyotype (46,XY). At the age of 2 years the proband had chest X-ray and the MRI of vertebral column which disclosed the presence of butterfly vertebra of Th2 and Th4. Upon repeated ECHO examination the PFO tended to be less and less haemodynamically significant (at the age of 2 years only trace PFO was noted). Endocrinological assays done between 2nd and 4th years of age showed normal levels of growth hormone, but at 4 years of age subclinical hypothyroidism was diagnosed. Holter exam performed at age 5 years and 4 months was unremarkable. The boy was re-investigated in the genetic clinic at the age of 8 years and 4 months. Dysmorphic features seen during his first evaluation were still conspicuous. His body measurements were as follows: height 120.8 cm (below 3rd percentile), body weight 23.6 kg (10th-25th percentile). Bone age assessment based on left carpal X-ray was relevant to the metrical age, and the IGF-1 levels were unremarkable. The patient still presented with hypothyroidism and required a daily substitution of 25 μg L-thyroxine. Ophthalmologic exam except for right-sided choroidal and retinal coloboma revealed convergent strabismus and hypermetropic astigmatism of the right eye (+1.5 Dsph/-0.5 Dcyl ax 150° = 1.0), whereas visual acuity of the left eye was 1.0. Psychomotor development was apparently normal. Family history was non-contributory.

## Methods

### Cytogenetic and Fluorescent In Situ Hybridization (FISH) studies

Chromosomal analysis on the basis of GTG technique at 550 band resolution per haploid genome was performed on peripheral blood lymphocytes of the patient and his parents. FISH was carried out with use of 5p/5q subtelomeric probes (TelVysion probe 5p/5q, *Vysis*) according to the manufacturer’s protocols.

### Sequencing and MLPA

Genomic DNA was extracted from peripheral blood leukocytes according to salting-out method. The local ethics committee (Institutional Review Board at Poznan University of Medical Sciences) approved the study and written, informed consent was obtained from all participants or their legal guardians prior to genetic testing. The entire coding sequences of the *TBX5* (GenBank NM_000192) and *SALL4* (GenBank NM_020436) genes comprised of eight and four exons respectively, and the flanking intronic regions were amplified in PCR reactions and directly sequenced using dye-terminator chemistry (kit v.3, ABI 3130XL). Sequences of the primers used for amplification and sequencing PCR reactions are given in Tables 1 and 2. PCR conditions used for amplification of both genes were as follows: 40 cycles, denaturation in 95°C (30’), annealing (30’) with temperature starting from 63°C, decreasing to 55°C (touchdown PCR −0.2°C per cycle), and elongation in 72°C (45’). Multiplex ligation-dependent probe amplification (MLPA) for all exons of both *TBX5* and *SALL4* genes was performed by means of P180-B1 commercial kit according to the manufacturer’s protocol (MRC Holland).

**Table 1:** Sequences of the primers used for *TBX5 *gene (MIM ID*601620; GenBank NM_000192) amplification and sequencing

**Exon name (fragment)**	**Forward primer sequence 5**^**′**^**-3**^**′**^	**Reverse primer sequence 5**^**′**^**-3**^**′**^	**Product size (bp)**
TBX5_e2	TCTCTCTCTGTCCTCCCCAC	CAAGAGAAGCCGAGCAGG	310
TBX5_e3	GGGAAGGAATGCCCACTAC	TTCCaagccaccttttcttc	231
TBX5_e4	TTAAAATGGATGGAGGCTGC	TTTTGGGAGAAGGTTCCAC	256
TBX5_e5	GTGCAGTGCGCTACCTCC	GAAACCCagtgagaagaaggg	279
TBX5_e6	GGTTTTATCTGGAGACAAAGGG	CAGGAAAACCTTGCAGATTC	293
TBX5_e7	CATGTCCTGAGGTGGTCTTG	GTTGCTGCTGGCTTACCTG	232
TBX5_e8	TCAGCCACTCAGGAAATCTG	CCCCAACCCAAGGAAAGG	356
TBX5_e9	CTCCACTTTTAGCTGCCTGG	TAGATCAGCATCCAGCGACC	797

**Table 2:** Sequences of the primers used for *SALL4 *gene (MIM ID*607343; GenBank NM_020436) amplification and sequencing

**Exon name (fragment)**	**Forward primer sequence 5**^**′**^**-3**^**′**^	**Reverse primer sequence 5**^**′**^**-3**^**′**^	**Product size (bp)**
SALL4_e1	GGGGTAAATTTCCCAACTCC	GCGTACGTCCGGGAAGC	263
SALL4_e2(a)	atagatgtgagcgacggtgc	AAGGTCTTCAGAGTGTCGGC	729
SALL4_e2(b)	TGGTGCCAACAGCATCC	CACTTTGTCCTGGAACTCGG	730
SALL4_e2(c)	CTGTCTGTGGTCATCGCTTC	GCATCACGGCATTAGTGAAC	684
SALL4_e2(d)	AGACACACCTTGGGGTTCAC	TAAAGTTCAACCCAGGCTCC	708
SALL4_e3	AAAGATCTCTTTTGCTTTGAAGAG	TGCCAATAAGAAGACACCTGG	420
SALL4_e4	ATTCTTGGCTTGCCAGTGAG	TCGTGATTGTAGCACTTGCC	652

### Array comparative genomic hybridization (array CGH)

Array comparative genomic hybridization (array CGH) was carried out with the use of two independent microarrays: whole-genome 180 K oligonucleotide array (*Agilent*) and 135 k NimbleGen oligonucleotide CGX array (*Roche NimbleGen*) according to standard protocols provided by the manufacturers. In the first case, analysis was performed with Feature Extraction and CGH Analytics software (*Agilent*), with the following settings used: Aberration Algorithm: ADM-2; Threshold: 6.0; Window Size: 0.2 Mb; Filter: 5 probes, max log2ratio = 0.29. In the second case, analysis was done with NimbleScan (*Roche NimbleGen*) and Genoglypix® software (*Signature Genomics*).

### Quantitative real-time PCR (qPCR)

To independently test for a number of 5q35.2-5q35.3 copies we developed a qPCR assay. We used a set of five primer pairs, three of which were located within the duplicated region and two lying centromeric against the duplication start point. qPCR was performed in a total volume of 12 μl in each well containing 6 μl of SYBR Green PCR Master Mix (*Applied Biosystems*), 5 μl of genomic DNA (1 ng/ml), and 1 μl of primers (0.2 mmol each). All samples were run in triplicate in separate wells to allow for the quantification of the target sequences normalized to *Albumin* (*ALB*)*.* PCR conditions were as follows: initial denaturation step at 95°C followed by 40 cycles (denaturation at 95°C for 15 s, annealing with elongation at 60°C for 1 min). By use of calibrator DNA derived of a normal healthy control, the gene copy number was measured on the basis of the comparative DD*C*t method. In addition, we performed a sex determination for the individuals, calculating the *Factor VIII* (*F8*) exon 3 relative to our endogenous control *Albumin,* to assure its reliability. Primer sequences for *ALB* and *F8* were as follows: *ALB_F* - tgttgcatgagaaaacgcca, *ALB_R* - gtcgcctgttcaccaaggat; *F8_F* - gccaagaagcatcctaaaacttg, *F8_R* - ggcgaggactaagggagcat.

### Additional probands with bilateral radial aplasia

To identify further probands and test the frequency of 5q35 duplication we screened a cohort of another 8 unrelated sporadic patients (6 males and 2 females) with bilateral radial agenesis and absent thumbs. All patients were of Polish ethnicity and were seen in our genetic clinic at different ages varying from 3 months to 31 years. Four cases manifested isolated limb malformation, whereas the other four presented with additional features comprising congenital heart defect (3 cases), and mental retardation (1 case). The patients were prescreened by bi-directional sequencing and MLPA for *TBX5* and *SALL4* mutations and turned out to be negative. Additionally, a patient affected by mental retardation was analyzed by means of GTG banding, which showed normal result.

## Case presentation

**Figure 2:**
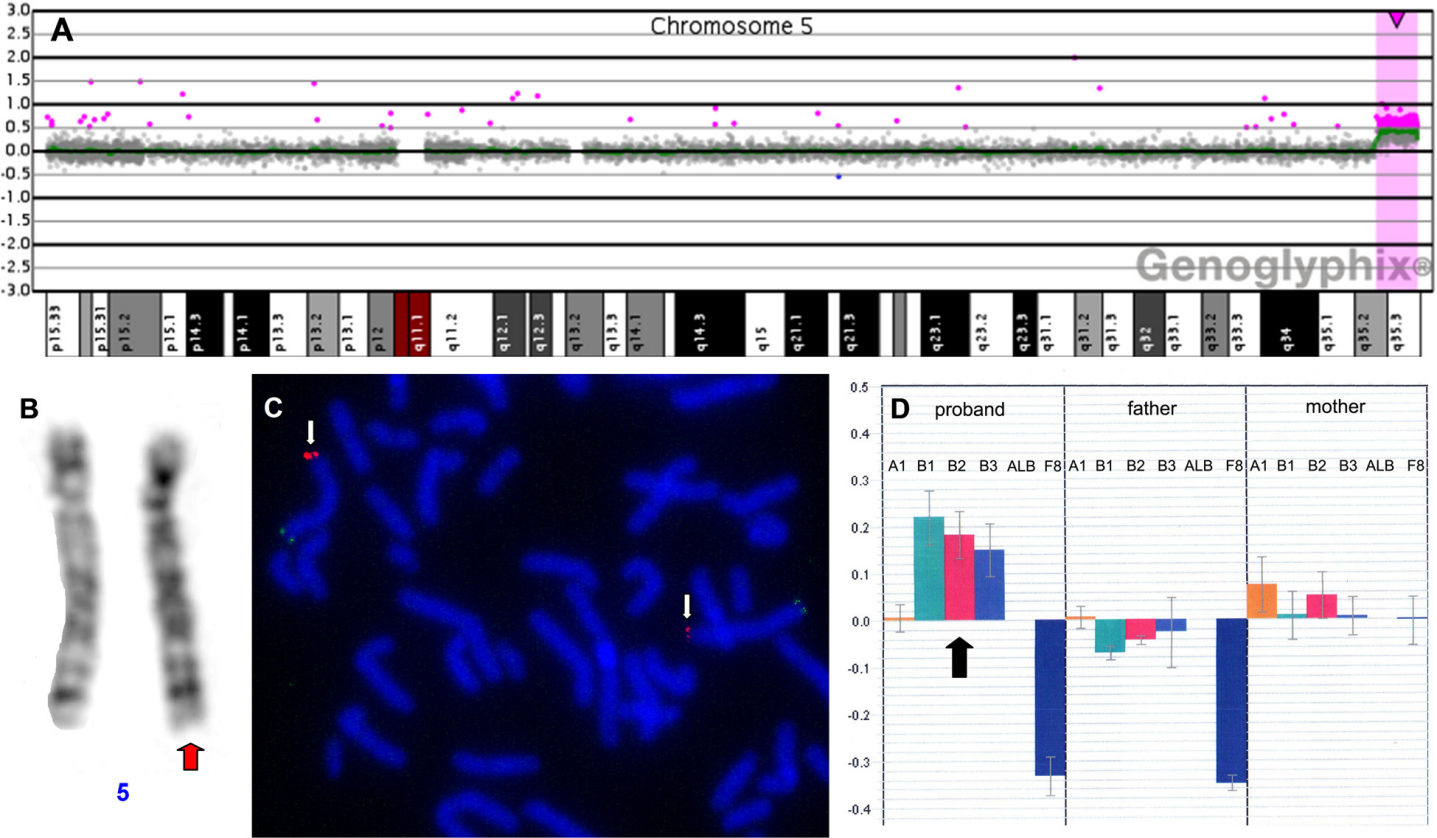
**Results of molecular and cytogenetic laboratory tests. A**) Array CGH profile of chromosome 5 showing an excess of material deriving from 5q35.2-5q35.3 in the proband **B**) Partial karyotype showing proband’s chromosomes 5; possible excess of material on 5q was indicated by red arrow **C**) FISH analysis with subtelomeric probes (*Vysis*) for 5q (red signal) and 5p (green signal) showing the location of the extra material on 5q (red signal was indicated by white arrows) **D**) qPCR results for 5q35.2-q35.3 segment performed in the proband and both parents: three highly specific primer pairs confirmed the presence of three copies in the proband (indicated by black arrow) and showed two copies in both parents.

Chromosomal analysis done on peripheral blood lymphocytes (conventional GTG banding at 550 band resolution) was unremarkable in the proband as well as in both parents. Differential diagnosis in the proband included Holt-Oram and Okihiro syndromes, therefore molecular screening of *TBX5* and *SALL4* genes was performed by means of sequencing and MLPA. The study ruled out the presence of point mutations and intragenic copy number changes within the coding portion of both genes. Array CGH of proband’s DNA detected a duplication on 5q35.2-5q35.3 between positions 175243241–180645010 (*Agilent* platform) and 175234642–180619169 (*Roche NimbleGen* platform) according to HG18 (for schematic view of Roche aCGH results see Figure 2A). Next, qPCR with a set of primers highly specific for 5q35.2-5q35.3 showed three copies of the region of interest in the proband, hence confirming array CGH results. Two copies of the investigated region were found in both parents (Figure 2D; for primer sequences and coordinates see Table 3). The same qPCR assay was used to study a cohort of 8 non-consanguineous probands with radial agenesis (with or without heart defect) negative for *TBX5* and *SALL4* mutations. qPCR test revealed no copy number change in any of the probands. Retrospective analysis of GTG-banded chromosomes identified possible gain on 5q (Figure 2B), which could be easily missed without array CGH results. FISH with 5p/5q subtelomeric probes was used on metaphase and interphase chromosomes to localize the extra copy of distal 5q. The signals from 5q FISH probe were detected on chromosome 5 in the proband as well as in both parents, thus confirming that the duplication did not result from parental chromosomal translocation (Figure 2C).

**Table 3:** Sequences of the primers used for quantifying the number of copies of 5q35.2-5q35.3, along with coordinates for each amplified genomic region (HG18), its relative position with regard to the start point of the duplication, and the number of copies detected in the proband

**Primer name**	**Primer sequence**	**Chromosomal band**	**Coordinates of the amplicon (HG18)**	**No of copies detected in the proband**	**Position of the amplicon in ref. to the start of the duplication**
5q35.2_A1_F	AACAGGCTCACGCCTTCTTA	5q35.2	175182611 - 175182696	2	- 61 kb
5q35.2_ A1_R	GAAGCACCCAACACATCCTT				
5q35.2_ A2_F	CCTTACCAGCAGGGACACAT	5q35.2	175201237 - 175201316	2	- 42 kb
5q35.2_ A2_R	CATGGCCCTCCTACACATCT				
5q35.3_B1_F	GTGGAATGATCACGATGCTG	5q35.3	175286270 - 175286357	**3**	+ 43 kb
5q35.3_ B1_R	TGCACAGTCCAACAGACACA				
5q35.3_ B2_F	CTGCTCAGCGGGATCTATGT	5q35.3	176356609 - 176356696	**3**	+ 1,12 Mb
5q35.3_ B2_R	TCAAGTCTGCCCCAACTCTT				
5q35.3_ B3_F	CCAATCCTGGCATGAGACTT	5q35.3	179355063 - 179355146	**3**	+ 4,12 Mb
5q35.3_ B3_R	GCAAACTGTGTGGGAATCCT				

**Figure 3:**
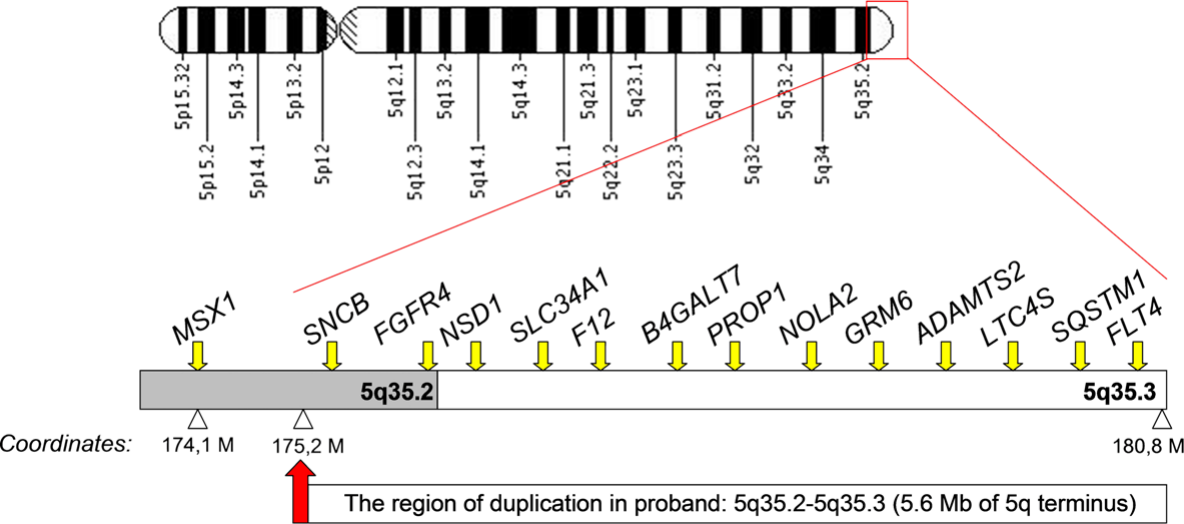
**An ideogram of the chromosome 5 with schematic representation of the duplication identified in our proband.** All 13 disease-associated genes are listed above the sketch representing 5q terminus.

According to *Map Viewer* browser the duplicated region contains 154 genes, including 52 OMIM genes, out of which 13 are known to be disease causing (see Figure 3). The most proximal disease associated gene lying outside the duplication (around 1.1 Mb centromeric from the duplicated segment) is a homeobox containing gene *MSX2* also known as *HOX8*.

## Discussion

Our patient carries the smallest pure distal duplication of chromosome 5q (i.e. terminal part of 5q35.2 band and the entire 5q35.3 band). The phenotype of our patient showed significant overlap with Hunter-McAlpine syndrome, with such common features as short stature, heart defect, cryptorchidism, and craniofacial dysmorphism including prominent widened nasal bridge, almond-shaped eyes, thin vermillion of upper lip, and low-set dysplastic ears. In addition, our patient manifested less frequent symptoms (i.e. hypothyroidism) or even unique findings such as bilateral radial agenesis with absent thumbs, bilateral ulnar hypoplasia, abnormal biliary vesicle, and unilateral choroidal and retinal coloboma. Interestingly, absent thumbs were reported so far only in a single patient with an atypical copy number variation (CNV) on 5q, which was the interstitial triplication of the distal 5q segment encompassing *MSX2* (6.56 Mb, coordinates according to HG18: 173897858–180456069). Since absent thumbs were never noted in cases with a distal 5q duplication, the authors hypothesized that the relatively severe limb malformation was due to the increased dosage of 5q copies [9]. Although caused solely by a pure duplication, the skeletal phenotype in our patient was more severe and involved bilateral radial aplasia with completely absent Ist digital rays (thumbs and Ist metacarpals), and ulnar hypoplasia with bowing. The defect seen in our patient as well as in the former case can be both categorized to radial ray deficiency spectrum. We therefore suggest that limb malformation presented by our index was a more severe manifestation of a common defect, namely radial ray deficiency. Of note, the chromosomal microduplication identified in our proband (terminal 5.4-5.6 Mb) did not encompass the *MSX2* gene, which is located 1.1 Mb centromeric relative to its beginning. The *MSX* family, comprises *MSX1* and *MSX2* homeobox containing genes, which are important developmental regulators involved in the processes of limb, craniofacial, and ectoderm formation in vertebrates [10]. For example, *MSX1* is essential for tooth and facial bone development and its mutations lead to Witkop syndrome also known as nail dysplasia with hypodontia [11]. Moreover, duplications of *MSX2* cause Boston type craniosynostosis [6,12], whereas intragenic alterations or gene deletions result in parietal foramina, a disorder of deficient ossification of the skull [13,14]. It has been therefore postulated that duplications of *MSX2* are responsible for craniosynostosis and brachydactyly in Hunter-McAlpine patients. Thus far, 5q distal trisomy has never been associated with the absence of digits. However, based on the observation of a 5q tetrasomy carrying patient, it has been hypothesized that multiple copies of 5q (including *MSX2*) result in a more severe skeletal anomaly such as absent thumbs. Clinical manifestation of our proband and the underlying genetic defect show that in the case of 5q gain, an extra copy (copies) of *MSX2* is not necessary to give rise to a severe limb phenotype involving not only absent thumbs, but also bilateral radial aplasia and hypoplastic ulnae. Importantly, the size of the duplication in our case was smaller than that reported for other trisomy 5q patients and mapped 1.1 Mb telomeric to the *MSX2*. A plausible candidate gene causative for the limb malformation in our proband could be *FGFR4*. This gene is duplicated in our patient and maps around 1.3 Mb from the beginning of CNV. *FGFR4* encodes for a protein, which is a type 4 receptor for fibroblast growth factors (FGFs). Members of FGF protein family are involved in FGF signalling pathway and play an important role during limb development. FGF4 along with FGF8 is secreted by apical ectodermal ridge (AER) which maintains the FGF10 signal and induces proliferation in the mesoderm [15,16]. For example, loss of both *Fgf4* and *Fgf8* in mice is thought to result in a reduction of the proliferation rate in distal mesenchyme, followed by downregulation of *Fgf10* and premature degeneration of AER. Hence, in the absence of both *Fgf4* and *Fgf8*, increased mesenchymal cell death results in a reduction in limb bud size [17]. So far there has been no report on radial agenesis and absent thumbs in other patients carrying 5q duplication encompassing *FGFR4*, suggesting that an extra copy of this gene is not sufficient to give rise to the limb phenotype. Noteworthy, *FGFR4* maps about 1.3 Mb from the beginning of the duplication detected in our proband, which is relatively the closest position to the chromosomal breakage site for all known 5q duplications. Since both the limb malformation as well as the underlying genetic defect are unique in our patient we propose that a position effect resulting in altered long-range regulation of the *FGFR4* (or possibly *MSX2*) may be the underlying patomechanism for limb malformation in both our and 5q tetrasomy patient. Alternatively, an extra copy and/or dysregulation of another gene may be responsible for radial agenesis.

The high frequency of congenital heart abnormalities in patients with a 5q trisomy was attributed to the altered dosage of one or two cardiac developmental genes, *NKX2*-5 and *CSX1*, both mapping to chromosome 5q34 [18,19]. Interestingly, patient with the smallest pure distal duplication of 5q (encompassing terminal 6.4 of 5q35.2-q35.3) described to date in the literature, did not show any cardiac abnormality. This pointed to an observation that the direct duplication of 5q35.2-q35.3 may not lead to a cardiac phenotype [8]. In contrast, our patient carried even smaller distal duplication of 5q, however presented with a complex congenital heart defect including dextrocardia, dextroversion, and PFO. This may suggest that not only *NKX2*-5 or *CSX1*, but also other genes or regulatory elements located in distal 5q play an important role in the process of embryonic heart formation.

A duplication of the distal arm of chromosome 5q is known to be associated with short stature and microcephaly, and an increased dosage of *NSD1* gene was proposed to be responsible for a combination of these two features [8]. Deletions and point mutations of the *NSD1* gene cause Sotos syndrome with cerebral gigantism, overgrowth and macrocephaly [20]. It is theoretically possible, as suggested by Chen et al. [8], that dosage changes (decrease or increase) of *NSD1* lead to opposite phenotypes. Nonetheless, our patient had a duplication encompassing *NSD1* but although short statured, he did not presented with a microcephaly. This may be explained by the incomplete penetrance of the candidate microcephaly gene.

## Conclusions

In conclusion, we postulate that a terminal distal trisomy of 5q35.2-5q35.3, which maps 1.1 Mb telomeric to the *MSX2* gene is causative for both radial agenesis and complex heart defect in our proband. Although duplications of *MSX2*, a highly conserved developmental gene which plays a major role in cardiac and bone morphogenesis were considered responsible for at least some skeletal symptoms (including limb malformations) in 5q trisomy patients, we provide evidence that even more distally located duplications may give rise to a more severe limb phenotype. Based on this observation, we propose that other genes or altered *FGFR4* (or possibly *MSX2*) long range regulation contribute to the development of radial agenesis and absent thumbs.

Finally, we studied a small cohort of 8 unrelated probands manifesting bilateral radial agenesis with or without heart defect, who were negative for *TBX5* and *SALL4* mutations. None of them had 5q duplication detected upon qPCR, suggesting that this kind of molecular defect is not a common cause of radial agenesis and associated limb phenotype.

## Consent

Written informed consent was obtained from the patient’s parents to take part in the study as well as for publication of the images (including full-face pictures). A copy of the written consent is available for review by the Editor-in-Chief of this journal. Informed consent was obtained from 8 unrelated probands or their legal guardians to participate, and include details in the manuscript.

## Abbreviations

AER: Apical ectodermal ridge; ASD: Atrial septal defect; CGH: Comparative genomic hybridization; CNV: Copy number variation; LVNC: Left ventricular noncompaction; PFO: Persistent foramen ovale; qPCR: Quantitative polymerase chain reaction; VSD: Ventricular septal defect.

## Competing interests

The authors declare no competing interests.

## Authors’ contributions

AJ, consulted the family, conceived the manuscript, performed molecular testing; AS, performed molecular testing of the patients and parents; DS, performed GTG banding and FISH studies; TT, consulted the family of interest; MJB, critically revised the manuscript; ALB, critically revised the manuscript. All authors read and approved the final manuscript.

## Pre-publication history

The pre-publication history for this paper can be accessed here:

http://www.biomedcentral.com/1471-2350/14/13/prepub
